# Neuroendocrine regulation of gonadotropin secretion in seasonally breeding birds

**DOI:** 10.3389/fnins.2013.00038

**Published:** 2013-03-25

**Authors:** Takayoshi Ubuka, George E. Bentley, Kazuyoshi Tsutsui

**Affiliations:** ^1^Department of Biology, Center for Medical Life Science, Waseda UniversityShinjuku, Tokyo, Japan; ^2^Department of Biology, College of Liberal Arts and Sciences, Tokyo Medical and Dental UniversityIchikawa, Japan; ^3^Department of Integrative Biology and Helen Wills Neuroscience Institute, University of California at BerkeleyBerkeley, CA, USA

**Keywords:** seasonal reproduction, hypothalamus-pituitary-gonadal axis, gonadotropins, gonadotropin-releasing hormone, gonadotropin-inhibitory hormone, thyroid hormone, melatonin, stress

## Abstract

Seasonally breeding birds detect environmental signals, such as light, temperature, food availability, and presence of mates to time reproduction. Hypothalamic neurons integrate external and internal signals, and regulate reproduction by releasing neurohormones to the pituitary gland. The pituitary gland synthesizes and releases gonadotropins which in turn act on the gonads to stimulate gametogenesis and sex steroid secretion. Accordingly, how gonadotropin secretion is controlled by the hypothalamus is key to our understanding of the mechanisms of seasonal reproduction. A hypothalamic neuropeptide, gonadotropin-releasing hormone (GnRH), activates reproduction by stimulating gonadotropin synthesis and release. Another hypothalamic neuropeptide, gonadotropin-inhibitory hormone (GnIH), inhibits gonadotropin synthesis and release directly by acting on the pituitary gland or indirectly by decreasing the activity of GnRH neurons. Therefore, the next step to understand seasonal reproduction is to investigate how the activities of GnRH and GnIH neurons in the hypothalamus and their receptors in the pituitary gland are regulated by external and internal signals. It is possible that locally-produced triiodothyronine resulting from the action of type 2 iodothyronine deiodinase on thyroxine stimulates the release of gonadotropins, perhaps by action on GnRH neurons. The function of GnRH neurons is also regulated by transcription of the GnRH gene. Melatonin, a nocturnal hormone, stimulates the synthesis and release of GnIH and GnIH may therefore regulate a daily rhythm of gonadotropin secretion. GnIH may also temporally suppress gonadotropin secretion when environmental conditions are unfavorable. Environmental and social milieus fluctuate seasonally in the wild. Accordingly, complex interactions of various neuronal and hormonal systems need to be considered if we are to understand the mechanisms underlying seasonal reproduction.

## Introduction

**Figure 1:**
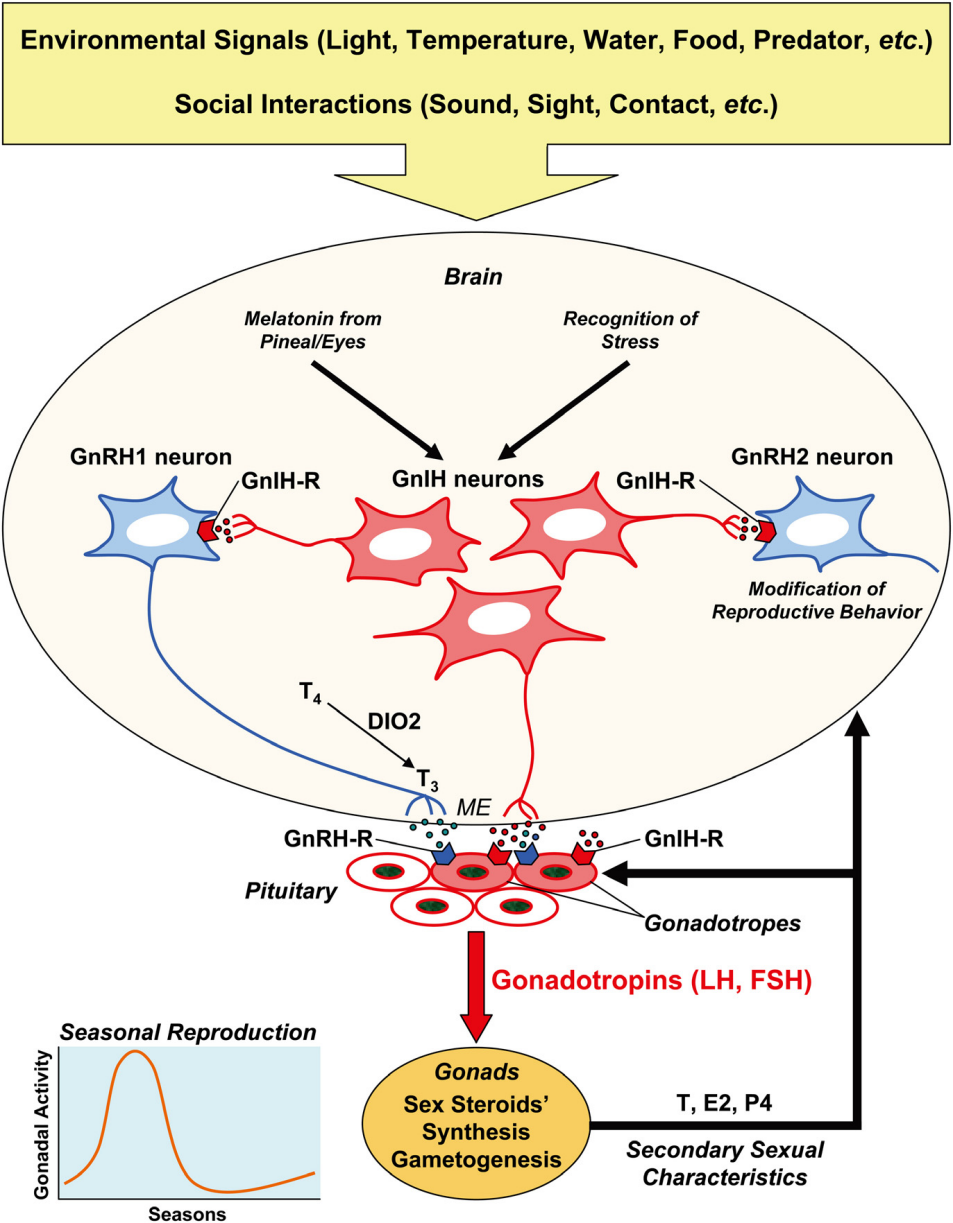
**Schematic model of the mechanism of seasonal reproduction in birds.** Seasonal breeding birds detect environmental signals, such as light, temperature, water, and food availability, predator pressure, etc. to time reproduction. Their reproductive activity is also shaped by social interactions with the conspecifics, in terms of sound, sight, contact, etc. Hypothalamic neurons in the bran integrate external and internal signals, and regulate reproduction by releasing neurohormones at the median eminence (ME) to regulate the pituitary hormone secretion. The anterior pituitary gland synthesizes and releases gonadotropins (LH, FSH), and gonadotropins act on the gonads to stimulate gametogenesis and sex steroid secretion. Sex steroids induce secondary sexual characteristics of the organism. A hypothalamic neuropeptide, GnRH1, activates reproduction by stimulating gonadotropin synthesis and release *via* its receptor (GnRH-R) expressed on the gonadotropes. Another hypothalamic neuropeptide, GnIH, may inhibit gonadotropin synthesis and release directly by acting on the pituitary or indirectly by decreasing the activity of GnRH1 neurons *via* its receptor (GnIH-R) expressed on the gonadotropes and GnRH neurons. GnRH2 neurons may stimulate reproductive behavior, and GnIH may inhibit reproductive behavior by inhibiting GnRH2 neurons. GnRH and GnIH neurons in the hypothalamus and their receptors in the pituitary are also regulated by internal signals, such as testosterone (T), estradiol (E2), and progesterone (P4). It is hypothesized that locally produced triiodothyronine (T_3_) from thyroxine (T_4_) by the action of type 2 iodothyronine deiodinase (DIO2) at the mediobasal hypothalamus stimulates the release of GnRH1. The function of GnRH1 neurons is also regulated by transcription of the GnRH gene. Because melatonin, a nocturnal hormone, stimulates the synthesis and release of GnIH in birds, GnIH may regulate daily rhythm of gonadotropin secretion. GnIH may also temporally suppress gonadotropin secretion when environmental or social condition is unfavorable.

Seasonal changes in reproductive physiology and behavior of birds are under hypothalamic control of pituitary gonadotropin (luteinizing hormone, LH; follicle-stimulating hormone, FSH) secretion. Gonadotropins secreted from the anterior pituitary gland stimulate gametogenesis (spermatogenesis, oogenesis) and sex steroid (androgens, estrogens, progestogens) synthesis in the gonad. Gonadal steroids induce the development of secondary sexual characteristics and facilitate reproductive behavior. A hypothalamic neuropeptide, gonadotropin-releasing hormone (GnRH), activates reproduction by stimulating gonadotropin synthesis and release. Another hypothalamic neuropeptide, gonadotropin-inhibitory hormone (GnIH), inhibits gonadotropin synthesis and release directly by acting on the pituitary gland or indirectly by decreasing the activity of GnRH neurons. This review attempts to understand how GnRH and GnIH neuronal systems can integrate external and internal environmental information and regulate gonadotropin secretion to time seasonal reproduction in birds (Figure 1).

## Seasonal reproduction

### Environmental information according to season

Birds time their breeding so that young are hatched when there is enough food for them to be raised (Lack, 1968). Seasonal breeding is thought to be regulated by two sets of environmental factors, “ultimate” and “proximate” factors (Baker, 1938). The most important ultimate factor is thought to be the availability of an adequate food supply for the mother during the final stages of ovarian development as well as for the hatchlings. Other ultimate factors may include nesting conditions, predation pressure, and climate factors. These ultimate factors are not necessarily the ones used to trigger and regulate the initial secretion of reproductive hormones, because it is necessary to anticipate the hatching date and to begin preparations for breeding ahead of the time when the offspring must be produced (Follett, 1984).

In birds living in mid and high latitudes the annual change in day length controls the timing of breeding; photoperiod is thus the predominant proximate factor for birds living in such regions (Follett, 1984). Other environmental factors can accelerate or retard photoperiodically-induced gonadal growth (Farner and Follett, 1979). These include rainfall (Leopold et al., 1976), ambient temperature (Perrins, 1973), and the presence of males for stimulating ovarian development (Marshall, 1936; Hinde, 1965; Cheng, 1979; Bentley and Ball, 2000). On the other hand, it is thought that photoperiod is not the proximate factor for many tropical and desert species (Marshall, 1970). The proximate factors used to time breeding in tropical birds remain largely unknown although correlative analyses suggest rainfall, territory, nest site availability, nest materials, and food supply are involved (Immelmann, 1971; Zann et al., 1995). The reproductive axis of tropical birds may remain in a state of “readiness to breed,” and full functionality may be triggered by the relevant proximate cues (Perfito et al., 2006).

### Photoperiod

The majority of temperate-zone passeriformes undergo dynamic seasonal changes in their reproductive activities. Gonadal development occurs in spring in response to increasing day length (“photostimulation”). However, the gonads are maintained in a functional condition only for a short period, and they spontaneously regress after extended exposure to long day lengths (“absolute photorefractoriness”). After becoming photorefractory, exposure to short day lengths is required for many passeriforme birds to regain “photosensitivity” and thus allow for photostimulation again (Wingfield and Farner, 1980; Dawson et al., 2001). Note that absolute photorefractoriness occurs when day lengths are still increasing.

A condition similar to absolute photorefractoriness is “relative photorefractoriness.” Even if relative photorefractoriness has been induced and the gonads have regressed, a subsequent substantial increase in day length will initiate reproductive maturation without the need for a short day length “sensitization” in some birds, such as Japanese quail (Robinson and Follett, 1982). If quail experience day lengths of over 11.5 h, rapid gonadal development occurs. After about 3 months and when day length decreases below 14.5 h of light per day (in the wild) complete gonadal regression occurs (Nicholls et al., 1988). However, if the day length is subsequently artificially increased further, a full return to reproductive maturity occurs. If quail are maintained on any constant long day length, no form of photorefractoriness will be elicited unless they experience a decrease in day length, for example, from 23 h of light per day to 16 h (Nicholls et al., 1988).

Song sparrows and house sparrows show characteristics of both absolute and relative photorefractoriness (Dawson et al., 2001). Song sparrows (Wingfield, 1983) and house sparrows (Dawson, 1998a) eventually become photorefractory during exposure to long photoperiods, but the timing can vary widely among individuals. If, in a laboratory environment, the length of the long day is decreased slightly, gonadal regression occurs sooner and is more synchronized among individuals. Song sparrows can show renewed gonadal maturation without the exposure to short photoperiods to “break” photorefractoriness and induce photosensitivity that is required in absolutely photorefractory species (Wingfield, 1993).

### Photoreceptor

It is thought that light can affect the behavior and physiology of birds via three different pathways: the eyes, the pineal, and the deep brain (Underwood et al., 2001). The avian eye is not only a functional photoreceptor, but also contains a circadian clock(s) and produces a circadian rhythm of melatonin secretion (Binkley et al., 1980; Hamm and Menaker, 1980). The principal cells within the pineal of most non-mammalian vertebrates have characteristics of a photosensory cell, including the presence of an outer segment composed of stacked disks containing photopigments (Collin and Oksche, 1981). The avian pineal organ is photosensitive and it is also the locus of a circadian pacemaker (Takahashi and Menaker, 1984; Okano and Fukada, 2001). The location of the extrapineal–extraretinal photoreceptors mediating circadian entrainment in birds has not been established. In a study using opsin antibodies, cerebrospinal fluid-contacting cells were labeled in the septal and the tuberal areas in the ring dove (Silver et al., 1988), and lateral septum in the pigeon (Wada et al., 2000). Light and confocal microscopy revealed interactions of GnRH-immunoreactive (-ir) and opsin-ir materials in the preoptic area (POA) and in the median eminence, suggesting a direct communication between these putative deep brain photoreceptors and GnRH neurons (Saldanha et al., 2001). Recently, Nakane et al. (2010) have reported that Opsin 5 (OPN5; also known as GPR136, Neuropsin, PGR12, and TMEM13)-ir neurons in the paraventricular organ (PVO) contact the cerebrospinal fluid and extend their fibers to the external zone of the median eminence adjacent to the pars tuberalis of the pituitary gland, which may translate photoperiodic information into neuroendocrine responses (Nakane et al., 2010).

### Measurement of daylength

The circadian system is involved in birds' measurement of day length. In a classic experiment, white-crowned sparrows held on short day lengths [8 h light (8L):16 h dark (16D)] were placed in continuous darkness. When birds were exposed to a single 8 h photophase (light period), an increase in LH occurred only if the photophase coincided with a time period 12–20 h after the subjective dawn (Follett et al., 1974). There are two possible models of how circadian rhythms might be involved in photoperiodic time measurement in birds (Goldman, 2001). The “external coincidence model” assumes that the organism possesses a circadian rhythm of “photosensitivity.” If coincidence between this rhythm and light occurs under long days, it induces gonadotropin secretion. The “internal coincidence model” assumes that the induction occurs when coincidence is established between two separate circadian oscillators (dawn and dusk oscillators). As a consequence of using a circadian clock for photoperiodic time measurement, light is not required throughout the day to induce gonadal growth, but pulses of light simulating dawn and dusk can cause induction if one of the pulses coincides with the phase of photosensitivity (Follett, 1973). If 15 min pulses of light were given at different times in the night to quail housed on a light:dark cycle of 6L:18D, induction occurs only if the pulses are within 12 to 16 h after dawn (Follett and Sharp, 1969).

Light intensity can also modify the reproductive responses of birds under the same photoperiod (Bissonnette, 1931; Bartholomew, 1949). Bentley et al. (1998) showed that photosensitive starlings transferred from short days to long days of different light intensities underwent graded reproductive responses according to the light intensities they experienced. The growth in their testes size and the development of photorefractoriness were similar to those seen in starlings exposed to different photoperiods. These data contradict the external coincidence model in that light falling in the photoinducible phase should cause a long day response. However, this discrepancy might be explained by the possibility that low light intensities only weakly entrain the circadian oscillations of the photoinducible phase, so that light is experienced in only part of the photoinducible phase (Bentley et al., 1998).

### Biological clock and calendar

The circadian system of birds is composed of several interacting sites, including the pineal organ, the suprachiasmatic nucleus (SCN) of the hypothalamus, and eyes. Each of these sites may contain a circadian clock (Underwood et al., 2001). However, significant variation exists among birds in the relative roles that the pineal, the SCN, and the eyes play within the circadian system and influence circadian activity patterns. For example, in the house sparrow circadian pacemakers in the pineal play the predominant role, whereas in the pigeon circadian pacemakers in both the pineal and eyes play a significant role. In Japanese quail, ocular pacemakers play the predominant role. There has been controversy on the precise location of the avian homologs of the mammalian SCN. The medial hypothalamic nucleus (MHN, also termed the medial SCN) and the lateral hypothalamic retinorecipient nucleus (LHRN, also termed the visual SCN) are the possible homologs of the mammalian SCN (Cassone and Moore, 1987; Norgren and Silver, 1989, 1990; Shimizu et al., 1994).

A self-sustaining circadian oscillation in the cells of clock structures is generated by a transcription-translation feedback loop of clock genes, the presence of which appears to be a conserved property from fruit flies to humans. Clock and Period homologs (qClock, qPer2, and qPer3) were cloned in the Japanese quail. qPer2 and qPer3 showed robust circadian oscillations in the eye and in the pineal gland, although qClock mRNA was expressed throughout the day. qPer2 mRNA was induced by light, whereas qClock or qPer3 were not (Yoshimura et al., 2000). These clock genes were expressed in the MHN, but not in the LHRN in quail (Yoshimura et al., 2001). On the other hand, Per2 mRNA is expressed both in the MHN and in the LHRN with rhythmic expression patterns in the house sparrow (Abraham et al., 2002). Clock genes such as Per, Cry, Clock, Bmal, E4bp4 are all expressed and differentially oscillate in quail and chicken pineal gland (Doi et al., 2001; Okano et al., 2001; Yamamoto et al., 2001; Fukada and Okano, 2002; Yasuo et al., 2003). The avian pineal gland seems to possess a functional circadian oscillator composed of a transcription/translation-based autoregulatory feedback loop as in the mammalian SCN (Fukada and Okano, 2002). Thus, multiple oscillators are present in birds, and they are somehow coordinated to convey circadian rhythmicity.

Annual seasonal activities of birds, such as reproduction, molt, and migration can persist for many cycles with a period deviating from 12 months under constant conditions. These cycles have been named “circannual rhythms” (Gwinner, 2003), although many of them deviate significantly from a 12 month period. These cycles have been experimentally demonstrated for at least 20 species of birds under specific lighting conditions (Gwinner, 1986; Gwinner and Dittami, 1990; Guyomarc'h and Guyomarc'h, 1995). In European starlings, a cycle of photosensitive and photorefractory phases continues in constant photoperiods close to 12 h (Gwinner, 1996; Dawson, 1997). Under photoperiods longer than 12 h starlings remain in the photorefractory state, whereas under shorter photoperiods they remain in the photosensitive state (Gwinner, 1996). Castrated starlings exposed to 12L:12D did not exhibit cyclic rhythms of this type (Dawson and McNaughton, 1993). Accordingly, the reproductive cycle that was observed for intact birds under this specific constant photoperiod (12L:12D) might be generated as a result of complex interactions within the hypothalamus-pituitary-gonadal (HPG) axis, rather than as a result of a circannual “calendar” in the brain (Ubuka and Bentley, 2011).

### Melatonin

The pineal glands of all vertebrates show daily rhythms in the activity of the enzymes in the indolamine-synthesizing pathway that produces 5-methoxy-N-acetyltryptamine (melatonin). Plasma melatonin concentration is always higher at night in both diurnal and nocturnal animals. Melatonin is synthesized from serotonin by the action of N-acetyltransferase to N-acetylserotonin, and by hydroxyindole-O-methyltransferase (HIOMT) to melatonin. Rhythms of melatonin synthesis and release from the pineal gland have been shown in pigeons, house sparrows, quail, and chicken. Circadian rhythms in melatonin secretion from the cultured pineal gland can persist in constant condition and they can be entrained by 24 h light-dark cycles (Murakami et al., 1994; Barrett and Takahashi, 1997; Brandstätter et al., 2000, 2001). Significantly, the eyes can contribute up to 30% of plasma melatonin in pigeons and Japanese quail (Underwood et al., 1984; Oshima et al., 1989). The eyes of Japanese quail show a rhythm in melatonin content (high at night, low in the day) that can be entrained by light directed exclusively to the eyes (Underwood et al., 1990). Circadian pacemakers in the pineal and in the eyes are thought to communicate with the hypothalamic pacemakers via the rhythmic synthesis and release of melatonin (Chabot and Menaker, 1992; Underwood et al., 2001).

Three melatonin receptor subtypes, Mel1a, Mel1b, and Mel1c are identified in birds. These are G-protein-coupled receptors (GPCRs) (Reppert et al., 1995). Mel1a and Mel1c receptors are present in the LHRN (visual SCN) in the chicken brain (Reppert et al., 1995). Contrary to the melatonin rhythm, which is higher in the scotophase (dark period), [125I]-iodomelatonin binding in the brain is higher during the photophase and lower during the scotophase in chicken (Yuan and Pang, 1992), quail (Yuan and Pang, 1990), and pigeon (Yuan and Pang, 1991). Sex differences and the effect of photoperiod on [125I]-iodomelatonin binding were also observed in the avian brain. Density of melatonin binding sites was higher in males than in females in some telencephalic nuclei (e.g., HVC and Area X) of songbirds (zebra finch, house sparrow), and in the visual pathways and the POA in quail (Aste et al., 2001). Quail belonging to the short day (8L:16D) group expressed a significantly higher melatonin receptor density in the optic tectum and nucleus triangularis, while the long day (16L:8D) animals had a higher density of receptors in the hyperstriatum and nucleus preopticus dorsalis (Panzica et al., 1994). Recently, Bentley et al. (2012) have shown season- and context-dependent sex differences in melatonin receptor density in Area X of European starlings (Bentley et al., 2012).

Photoperiodic mammals rely on the annual cycle of changes in nocturnal secretion of melatonin to drive their reproductive responses (Bronson, 1990). In contrast, a dogma exists that birds do not use seasonal changes in melatonin secretion to time their reproductive effort and a role for melatonin in birds has remained enigmatic (Bentley, 2001). The effects of pinealectomy (Px), bilateral orbital enucleation (Ex), and Px plus Ex on seasonal regulation of reproduction were tested in tree sparrows, a highly photoperiodic species (Wilson, 1991). Although there was an accelerating effect of Ex on the changes in their testicular size, Px, Ex, and Px plus Ex birds revealed photostimulation, photorefractoriness, and recovery of photosensitivity in the same time course as in intact birds. The effects of artificial extension of the duration of circulating melatonin on reproductive status were tested using Japanese quail by exogenous melatonin injection (Juss et al., 1993). Male quail reared under non-stimulatory short days (8L:16D) were switched to 12L:12D and given daily melatonin injections at dusk (10 μg 2 h before dusk and 10 μg at dusk) or at dawn (10 μg 2 h before dawn and 10 μg at dawn) for 3 weeks. Contrary to the prediction, melatonin injection resulted in significant stimulation of LH concentration and testicular size. Despite the accepted dogma, there is evidence that melatonin is involved in the regulation of seasonal reproductive processes. Male Japanese quail reared under non-stimulatory short days (8L:16D) were used to test the effect of anti-melatonin antibody (anti-MEL) administration on the reproductive status (Ohta et al., 1989). Intravenous injection of anti-MEL just before lights-off for 3 days significantly increased testosterone (T) concentration and testicular size after 2 weeks even if the quail were kept under the same non-stimulatory photoperiod (8L:16D). There are also reports showing inhibitory effects of melatonin on seasonal recrudescence in quail (Guyomarc'h et al., 2001) and on LH secretion in chicken (Rozenboim et al., 2002). In a more recent study on wild great tits, melatonin administration significantly delayed the onset of egg-laying in spring (Greives et al., 2012).

### Temperature

Japanese quail do not show a complete decrease in levels of circulating LH concentrations or complete involution of the testes following transfer from long to short days without decreasing the temperature in the laboratory. Wada et al. (1990) have experimentally demonstrated that low ambient temperature together with the change of the photoperiod from long to short days was required to reduce circulating LH concentration to a non-breeding level at which the gonad and the accessory sex organs regressed completely. Photoperiodic changes from long to short days under moderate temperature resulted in a decrease in circulating LH to the basal breeding level that could maintain activity of the gonads and the accessory sex organs. These results suggest that ambient temperature can influence the timing of termination of reproductive activity in Japanese quail (Wada et al., 1990; Tsuyoshi and Wada, 1992).

Kobayashi et al. (2004) investigated the effect of short day length and low temperature on pituitary mRNA levels of LHβ and common α subunit in male Japanese quail under natural and laboratory conditions. When birds were kept in outdoor cages under natural conditions at Waseda University in Tokyo [35° 42′N, 139° 43′E; monthly mean atmospheric temperature: 27.4°C in August (highest), 6.1°C in January (lowest)], both LHβ and common α mRNA levels decreased rapidly from August to September, and after a period of low levels from October through January, they began to increase in February and continued to increase until July. Plasma LH concentration followed similar changes in quail housed in outdoor cages in Tokyo (Wada et al., 1992). When birds were kept in the laboratory and transferred from long day length (16L:8D) at 20°C (LD20) to short day length (8L:16D) at 20°C (SD20) or 9°C (SD9) for 14 days, although pituitary LHβ and common α mRNA levels, plasma LH concentration, and testicular weight decreased significantly in SD9, their decrease was moderate in SD20. However, low temperatures under long days did not induce any significant change in these parameters (Kobayashi et al., 2004).

Wingfield et al. (2003) have investigated the effects of temperature on photoperiodically induced reproductive development and regression in mountain white-crowned sparrows (*Z. l. oriantha).* Captive populations of mountain white-crowned sparrows showed robust gonadal development following transfer to longs days (15L:9D), but low temperature (5°C) slowed down photoperiodically induced gonadal growth and subsequent regression, in both males and females. High temperature of 30°C tended to accelerate gonadal development and regression whereas gonadal development was intermediate in a group exposed to 20°C. Prior exposure to these temperature regimes while on short days (9L:15D) had no effect. However, there was no effect of temperature on photoperiodically induced rises in LH in either sex despite marked effects on gonadal growth (Wingfield et al., 2003). Dawson (2005) has investigated the effect of temperature on photoperiodically regulated gonadal maturation, regression and molt in starlings. Common starlings were kept in two indoor aviaries, in which photoperiod tracked natural changes, but temperature was held at either 20°C or 5°C (year 1), or at 18°C or 8°C (year 2). Although temperature had no effect on the rate or timing of testicular maturation, testicular regression occurred significantly earlier at the higher temperatures. Post-nuptial molt also started significantly earlier in both males and females (Dawson, 2005). These studies demonstrate that temperature has significant effects on reproduction also in songbirds.

### Social interaction

Considering the social nature of reproduction, it is not surprising that social interactions have dramatic effects on reproductive physiology and behavior in vertebrates (Hinde, 1965; Lehrman, 1965; Crews and Silver, 1985; Searcy, 1992; Wingfield et al., 1994; Maruska and Fernald, 2011). Male courtship behaviors can greatly enhance the development of reproductive physiology and behaviors of female birds. Bentley et al. (2000b) investigated the effects of conspecific and heterospecific song on reproductive development in domesticated (canaries) and wild songbirds (song sparrows). Although conspecific and heterospecific songs were equally effective in enhancing ovarian development, conspecific song induced oviposition earlier and at a greater frequency than in heterospecific and no song groups, with the fewest eggs being laid in the no song group (Bentley et al., 2000b).

### Seasonal change in gonadotropin secretion

Seasonal changes in reproductive activities are generally correlated with gonadotropin secretion. The primary effect of long day lengths on stimulating gonadotropin secretion has been shown in many birds, such as quail (Follett, 1976), white-crowned sparrow (Wingfield and Farner, 1980), tree sparrow (Wilson and Follett, 1974), canary (Nicholls, 1974), duck (Balthazart et al., 1977), and starlings (Dawson and Goldsmith, 1983). If male quail are transferred from short day lengths to long day lengths, the levels of gonadotropins rise substantially during the first week of photostimulation. Testicular growth and steroidogenesis begin and maturity is reached in about 5 weeks (Follett and Robinson, 1980). Gonadal steroids also affect gonadotropin secretion by negative feedback (King et al., 1989a; Dunn and Sharp, 1999). Female quail also grow their ovaries as a result of increased gonadotropin secretion induced by long day lengths. Gonadotropin levels become basal after transferring quail from long days to short days (Gibson et al., 1975). On the contrary, in most of the temperate-zone Passeriform birds, which undergo spontaneous photorefractoriness after exposure to long days, gonadotropin secretion is diminished and gonadal collapse occurs after a species-specific number of long days (Follett, 1984).

Seasonal change in gonadotropin secretion continues in the absence of the gonads. Castrated or intact quail show an identical time-course in LH and FSH secretion under natural photoperiods over 2 consecutive years, the difference being that in summer the gonadotropin levels in castrated birds are higher, as a result of lack of negative feedback from gonadal steroids (Follett, 1984). A similar annual cycle of LH secretion was observed in the plasma of intact and castrated white-crowned sparrows (Mattocks et al., 1976). It is also thought that gonads are not required for photorefractoriness to develop, because castrated white-crowned sparrows (Wingfield et al., 1980), canaries (Storey et al., 1980), and starlings (Dawson and Goldsmith, 1984) show a spontaneous fall in gonadotropin level under long days. Castration of photorefractory canaries does not cause enhanced LH secretion, but when photosensitivity is regained under short days there is an immediate rise in plasma LH (Nicholls and Storey, 1976).

## Role of GnRH and its receptor

Reproductive activities of vertebrates are primarily regulated by GnRH in the hypothalamus. This decapeptide was originally isolated from mammals (Matsuo et al., 1971; Burgus et al., 1972) and subsequently from chicken (King and Millar, 1982a,b; Miyamoto et al., 1982). The molecular structure of the originally isolated mammalian GnRH (mGnRH-I) is pEHWSYGLRPG-NH_2_. Chicken GnRH-I (cGnRH-I; pEHWSYGLQPG-NH_2_) differs by one amino acid from mGnRH-I, in that glutamine is substituted for arginine at position 8. Specific genes encoding the same cGnRH-I peptide were identified by cDNA cloning in Galliformes (chicken, quail, turkey) (Dunn et al., 1993; Kang et al., 2006), Anseriformes (goose, duck) (Huang et al., 2008), Columbiformes (dove) (Mantei et al., 2008), and Passeriformes (zebra finch, European starlings) (Stevenson et al., 2009; Ubuka and Bentley, 2009; Ubuka et al., 2009a). Existence of cGnRH-I peptide was also suggested in songbirds by high-performance liquid chromatography (HPLC) and cross-reactivity with various GnRH antisera (Sherwood et al., 1988). There is a second form of GnRH, which is called chicken GnRH-II (cGnRH-II). cGnRH-II was first found in chicken and subsequently in mammals (Miyamoto et al., 1984; King et al., 1989b; Morgan and Millar, 2004; Millar, 2005) and eventually in all vertebrate groups (Roch et al., 2011). The structure of cGnRH-II (pEHWSHGWYPG-NH_2_) differs by three amino acids from mGnRH-I or cGnRH-I at positions 5, 7, and 8. Most of the phylogenetic analyses support the existence of three GnRH clades in gnathostomes, named GnRH1, GnRH2, and GnRH3 (Tostivint, 2011). Depending on this classification, cGnRH-I and cGnRH-II belongs to GnRH1 and GnRH2 groups, respectively. Accordingly, we refer to cGnRH-I and cGnRH-II as GnRH1 and GnRH2, respectively, in this review (Figure 1).

Specific antibodies against avian GnRH peptides (GnRH1 and GnRH2) were made, and the histological localization of GnRHs was studied in chicken and quail (Mikami et al., 1988; van Gils et al., 1993). In mammals, the GnRH1 neurons originate at the olfactory placode and migrate to preoptic-septal nuclei during embryonic development (Schwanzel-Fukuda and Pfaff, 1989; Wray et al., 1989). The migration of GnRH1 neurons from the olfactory placode to the forebrain along the olfactory nerve was also observed in chickens (Norgren and Lehman, 1991; Akutsu et al., 1992; Yamamoto et al., 1996). In adult birds, GnRH1-ir cell bodies are found in a fairly wide area covering the POA to the thalamic region. On the contrary, magnocellular GnRH2-ir cell bodies were found in the area dorsomedial to the nervus oculomotorius in the midbrain. Fibers immunoreactive for GnRH1 or GnRH2 were widely distributed in the telencephalon, diencephalon, and mesencephalon (midbrain). In sharp contrast to the existence of abundant GnRH1-ir fibers in the external layer of the median eminence, GnRH2-ir fibers were absent or less prominent in this area, suggesting that the major GnRH controlling pituitary function is GnRH1 (Mikami et al., 1988; van Gils et al., 1993). Clerens et al. (2003) have isolated GnRH2-ir substances in the quail median eminence by high performance liquid chromatography (HPLC) and identified it by radioimmunoassay (RIA). The GnRH2-ir substances isolated by immunoaffinity chromatography, present in the quail median eminence extract, were also subjected to mass spectrometry. The immunoreactive peptide had a mass of 1235.56 Da, which is the same as synthetic GnRH2. In addition, MS/MS fragmentation generated an amino acid sequence corresponding to the sequence of GnRH2 (Clerens et al., 2003). This study unequivocally demonstrates the existence of GnRH2 in the median eminence of quail, although it may not be the major GnRH controlling pituitary function.

Specific RIAs for chicken LH (Follett et al., 1972), turkey LH (Burke et al., 1979), and chicken FSH (Scanes et al., 1977; Sakai and Ishii, 1980) have been developed and used to measure the effect of GnRH on gonadotropin release. The action of GnRH1 on LH release from chicken anterior pituitary cells was first shown *in vitro* (Millar and King, 1983). Subsequently, the effect of GnRH1 on LH and FSH release was shown both *in vivo* and *in vitro* in quail (Hattori et al., 1985). The activity of GnRH1 on LH release was more marked than that on FSH release both *in vivo* and *in vitro* (Hattori et al., 1985). The effect of GnRH2 on LH and FSH release was also shown *in vivo* and *in vitro* (Hattori et al., 1986). The activity of GnRH2 on LH and FSH release was almost equal to that of GnRH1. The action of GnRH2 on LH release was again more marked than on FSH release both *in vivo* and *in vitro*. No synergism was observed between GnRH1 and GnRH2 on LH or FSH release *in vitro* (Hattori et al., 1986), suggesting that these effects may be pharmacological acting on the same receptor.

The physiological roles of GnRH1 and GnRH2 on LH release were investigated in chickens (Sharp et al., 1990). The amount of GnRH1 in the median eminence was higher in laying than in out-of-lay hens, as measured by RIA. GnRH2 was not detected in the median eminence. The amount of GnRH1 in the hypothalamus increased in cockerels at the onset of puberty, but the amount of GnRH2 did not change. Active immunization of laying hens against GnRH1 but not against GnRH2 resulted in complete regression of the reproductive system. Accordingly, it was concluded that gonadotropin secretion in chickens is more likely to be controlled by GnRH1 (Sharp et al., 1990). On the other hand, GnRH2 may be involved in the control of sexual behaviors in various animals (Millar, 2003; Okubo and Nagahama, 2008). It was shown that GnRH2, but not GnRH1, administered to the brain increased copulation solicitation display, a female courtship behavior, in female white-crowned sparrows (Maney et al., 1997) (Figure 1).

Three GnRH receptor subtypes (type I, II, III) have been identified with distinct distributions and functions in vertebrates (Millar et al., 2004). These receptor subtypes belong to the GPCR superfamily. Two receptor subtypes were identified in chickens: type I (GnRH-R-I; Sun et al., 2001a,b) and type III (GnRH-R-III; Shimizu and Bédécarrats, 2006), according to the classification by Millar et al. (2004). GnRH-R-I is widely expressed, and GnRH2 has a higher binding affinity to this receptor and is more potent in stimulating accumulation of inositol trisphosphate, a secondary messenger molecule that can induce gonadotropin release, than GnRH1 (Sun et al., 2001a,b). Inositol trisphosphate accumulation in response to GnRH2 binding to GnRH-R-III was also more marked than in response to GnRH1. Because fully processed GnRH-R-III mRNA was exclusively expressed in the pituitary, and its mRNA level was positively correlated with reproductive states in both sexes, it is likely that GnRH-R-III plays a role in the regulation of gonadotropin secretion by pituitary gonadotropes (Shimizu and Bédécarrats, 2006). Despite the implication here that GnRH2 is more effective in regulation of gonadotrope function, the current opinion is that GnRH1, and not GnRH2, is the dominant regulator of gonadotropin release (Figure 1).

## Regulation of GnRH and GnRH-R

### By photoperiod

RIA and immunocytochemistry (ICC) using GnRH antisera have demonstrated cyclic changes in GnRH in songbirds in response to changing photoperiod. RIA revealed that hypothalamic GnRH content did not increase significantly during the first 6 weeks of photostimulation in female starlings in a laboratory environment. However, by 12 weeks after the onset of photostimulation, as birds became photorefractory, GnRH had decreased to levels significantly lower than those before photostimulation (Dawson et al., 1985). ICC with quantitative image analyses for both GnRH and its precursor, proGnRH-GAP, was performed during photoperiodically-induced reproductive cycle in male starlings (Parry et al., 1997). The size of cells immunoreactive for GnRH and proGnRH-GAP increased during gonadal maturation. A reduction in the number of cells immunoreactive for GnRH and the size of cells immunoreactive for both GnRH and proGnRH-GAP occurred during gonadal regression, though immunoreactivity for GnRH and proGnRH-GAP in the median eminence remained high. Immunoreactivity for GnRH and proGnRH-GAP was reduced significantly after gonadal regression. These observations suggest that photorefractoriness is promoted by a reduction in pro-GnRH-GAP and GnRH synthesis (Parry et al., 1997). Changes in GnRH1 in the POA and mediobasal hypothalamus (MBH) including the median eminence were measured in starlings during the recovery of photosensitivity under short days, and following photostimulation at various times during the recovery of photosensitivity (Dawson and Goldsmith, 1997). During exposure of long day photorefractory starlings to short days for 10 days, there was a significant increase in GnRH1 in the POA but not in the MBH. Photostimulation after 20 short days caused an immediate increase in GnRH1 in the POA, a delayed increase in the MBH, but no increase in plasma LH. Photostimulation after 30 short days caused an immediate increase in GnRH1 in the POA and the MBH and in plasma LH. These results show that the recovery of photosensitivity is gradual and the first measurable change occurs in the POA, consistent with photosensitivity being due to renewed GnRH1 synthesis (Dawson and Goldsmith, 1997). Recently, the complete sequence of GnRH1 precursor mRNA was identified in Passerine birds, European starlings (Stevenson et al., 2009; Ubuka et al., 2009a) and zebra finch (Stevenson et al., 2009; Ubuka and Bentley, 2009). The expression of GnRH1 precursor mRNA was found to be regulated as a function of age and reproductive condition in zebra finch (Ubuka and Bentley, 2009). In starlings, there was regulation of GnRH1 precursor mRNA expression as a function of season in birds exposed to natural photoperiod (Ubuka et al., 2009a) and in response to artificial changes in photoperiod (Stevenson et al., 2009). Photostimulation of chickens and turkeys also increased GnRH1 precursor mRNA expression (Dunn and Sharp, 1999; Kang et al., 2006).

### By thyroid hormone

It has been known for many decades that thyroid hormones can affect reproductive function, but the effects of thyroidectomy on seasonal breeding were often contradictory (Dawson et al., 2001). Gonadal regression caused by photorefractoriness is normally prevented by thyroidectomy, but in some studies, photorefractoriness appears to have been accelerated (Dawson and Thapliyal, 2001). Treatment with one of the thyroid hormones, thyroxine (T_4_), can mimic the effects of long photoperiods (Follett and Nicholls, 1988; Goldsmith and Nicholls, 1992; Wilson and Reinert, 1995). Reinert and Wilson (1996) argue in tree sparrows that the increase in plasma T_4_ concentrations following photostimulation (Sharp and Klandorf, 1981; Dawson, 1984) serves to program the subsequent gonadal cycle and molt. However, when Bentley et al. (1997) maintained T_4_ concentrations at subphysiological levels by treating thyroidectomized starlings held on long days with appropriate amounts of T_4_, these birds became photorefractory and molted. This result suggests that T_4_ simply has to be present for the appropriate responses to photoperiod to occur.

The site(s) of action of thyroid hormones may lie within the central nervous system since in starlings and sparrows, prevention of photorefractoriness by thyroidectomy is associated with maintenance of high levels of GnRH1, typical of photosensitive birds (Dawson et al., 1985; Reinert and Wilson, 1996; Dawson, 1998b). In addition, thyroidectomy of photorefractory birds results in an increase in GnRH1 (Dawson et al., 1986). Central administration of thyroid hormones to thyroidectomized tree sparrows restores all of the photoperiodic responses, with the effects of T_4_ being more potent than triiodothyronine, T_3_ (Wilson and Reinert, 2000). Chronic thyroidectomy appears to render starlings “photoperiodically blind,” because the GnRH response to an increase or a decrease in photoperiod is greatly attenuated (Bentley et al., 2000a), although less chronically thyroidectomized starlings still exhibit a robust long day photoperiodic response (Bentley et al., 1997). In house sparrows, the effects of thyroidectomy are very different. Again thyroidectomized birds appear to be photoperiodically blind; testicular size and GnRH1 are the same whether birds are on long or short photoperiods (Dawson, 1998b). However, although GnRH1 remains high, which is typical of photosensitive birds, testicular size remains minimal, which is typical of photorefractory birds. Accordingly, thyroid hormone may have different effects on GnRH1 synthesis and release, which may account for the apparently contradictory results on the effects of thyroidectomy on reproductive processes.

Recent molecular analyses suggest that local thyroid hormone activation in the hypothalamus may play a critical role in the regulation of seasonal reproduction in birds (Nakao et al., 2008b). If the light exposure occurs around 11–16 h after dawn (photoinducible phase), then the first detectable change in gonadotropin secretion begins around 22 h after dawn in Japanese quail (Follett and Sharp, 1969), and a wave of gonadotropin secretion occurs over the next few days (Follett et al., 1977; Nicholls et al., 1983). Meddle and Follett (1997) examined the effect of the first long day on expression of a transcription factor, Fos, in the basal tuberal hypothalamus, a brain area that includes GnRH1 neuronal terminals. Transfer of short day (6L:18D) quail to long days induced Fos-ir in neuronal cells in the infundibular nucleus and glial cells in the median eminence by 18 h of the first long day. This activation of specific brain areas was followed by the first rise in LH 20 h after dawn (Meddle and Follett, 1997). Fos-ir in the infundibular nucleus and LH release were also stimulated by subcutaneous N-methyl-D-aspartate administration in the white-crowned sparrow (Meddle et al., 1999). Yoshimura et al. (2003) hypothesized that important molecular events would be triggered in the MBH when light was given to quail during the photoinducible phase. Acute induction of type 2 iodothyronine deiodinase (DIO2) mRNA expression was observed in the ependymal cells of the MBH and in the infundibular nucleus by long-day treatments. DIO2 is an enzyme that catalyzes the conversion of T_4_ to T_3_, whereas type 3 iodothyronine deiodinase (DIO3) catalyzes the conversion of T_4_ and T_3_ to their inactive forms. Interestingly, the expression of DIO3 was down-regulated under long day conditions when DIO2 was up-regulated (Yasuo et al., 2005). Central administration of DIO2 inhibitor, iopanoic acid, reduced testicular growth of quail that were transferred from short to long day conditions (Yoshimura et al., 2003). T_3_ implantation to the MBH caused testicular growth and reduced encasement of nerve terminals by glial processes in the median eminence of quail (Yamamura et al., 2006). The glial processes do not physically occupy and block space in between axon terminals and capillaries of the portal system in the median eminence of photostimulated white-crowned sparrows, but they do in photorefractory birds (Bern et al., 1966; Mikami et al., 1970). Because GnRH nerve terminals are also in closer proximity to the basal lamina of the median eminence in long day quail (Yamamura et al., 2004), Yamamura et al. (2006) hypothesized a role for T_3_ in the regulation of photoperiodic GnRH secretion via neuro-glial plasticity in the median eminence.

In a more recent study, functional genomic analysis was performed using a chicken high-density oligonucleotide microarray during photoinduction in quail (Nakao et al., 2008a). The microarray detected two waves of gene expression in the MBH. Interestingly, thyrotropin β subunit (TSHβ ) mRNA expression peaked around 14 h after dawn, and up-regulation of DIO2 and down-regulation of DIO3 occurred around 18–19 h after dawn of the first long day. Spatio-temporal expression analyses of the genes revealed that the first wave events occurred in the pars tuberalis of the pituitary gland, whereas the second wave events occurred in the ependymal cells in the ventral walls of the third ventricle in the MBH. Glycoprotein α mRNA, which codes for the common subunit for TSH, LH, and FSH, was cyclically expressed in the pars tuberalis. TSH receptor was observed in the in the ependymal cells in the ventral walls of the third ventricle in the MBH, where the second wave genes were expressed, and the binding of [125I]-TSH was further observed. Intracerebroventricular administration of TSH induced the expression of DIO2, whereas anti-TSHβ antibody reduced DIO2 expression (Nakao et al., 2008a).

### By melatonin

What are the factors that induce TSHβ mRNA expression in pars tuberalis of short day quail by the first long day (Nakao et al., 2008a)? The removal of the inhibitory effect of melatonin on TSHβ mRNA expression may be a possible mechanism, because melatonin receptor (Mel1c) mRNA is expressed in the pars tuberalis in chicken (Kameda et al., 2002). Glycoprotein α is also expressed in pars tuberalis in chicken, and Px induces glycoprotein α subunit mRNA expression in the pars tuberalis (Kameda et al., 2002). Thus, local induction of thyroid hormones caused by changes in melatonin signaling might well be involved in at least the initial photoperiodic response. This may be one of the reasons why treatment with anti-melatonin just before lights-off for 3 days caused significant gonadal growth in quail kept in short day (8L:16D) condition (Ohta et al., 1989).

Although Px plus Ex treatment for 1 week eliminated melatonin concentration in the plasma, there was a substantial amount of melatonin-ir material remaining in the quail diencephalon, suggesting melatonin synthesis in the brain (Ubuka et al., 2005). Recently, a group of neurons that synthesize both dopamine and melatonin were identified in the premammillary nucleus of the turkey hypothalamus (Kang et al., 2007; El Halawani et al., 2009). These neurons express clock genes and a photoreceptor, melanopsin. Dopamine/melatonin neurons are activated when a light pulse is given during the photosensitive phase, associated with an up-regulation of GnRH1 mRNA expression. The expression of tryptophan hydroxylase 1 (serotonin synthesizing enzyme) mRNA level is low during the photophase and high during the scotophase, and tyrosine hydroxylase (rate limiting dopamine synthesizing enzyme) mRNA shows the opposite cycle. These hypothalamic dopamine/melatonin neurons may thus provide a novel role for melatonin in the regulation of seasonal reproductive cycles in birds (El Halawani et al., 2009). This finding may explain the lack of effect of Px and/or Ex on the avian reproductive system, because day length information could be encoded by the hypothalamic dopamine/melatonin neurons.

### By sex steroids

Effects of castration and estrogen administration to castrated birds on GnRH1 and GnRH-R-I mRNA expressions were studied in the cockerel (Sun et al., 2001a) (Figure 1). In juvenile cockerels, concentrations of GnRH1 mRNA in the POA increased after castration, and it was prevented by estrogen treatment. In the anterior pituitary gland, the concentration of GnRH-R-I mRNA increased after castration and it was reversed by estrogen treatment. On the other hand, in intact adult cockerels, estrogen treatment depressed plasma luteinizing hormone but did not affect concentrations of GnRH1 and GnRH-R-I mRNAs in the POA, basal hypothalamus, and anterior pituitary gland, suggesting that locally produced estrogen, by aromatization, may exert maximal suppression on GnRH1 and GnRH-R-I mRNAs (Sun et al., 2001a).

Effect of sex steroids on GnRH1 release from hypothalamic slices including medial basal hypothalamus and preoptic areas (MBH-POA) was studied in adult male Japanese quail (Li et al., 1994) (Figure 1). Short-term estradiol-17β (E2) exposure significantly potentiated norepinephrine (NE)-induced GnRH1 release. Neither T nor 5 alpha-dihydrotestosterone affected baseline or NE-stimulated GnRH1 release. On the contrary, pretreatment with E2 for 14 h in static culture before perifusion significantly reduced the NE-induced GnIH-I release. These data provide evidence for E2 modulation of GnRH1 release, which varies with exposure time (Li et al., 1994).

The mechanism whereby gonadal steroids modulate GnRH-stimulated LH secretion by primary cultures of chicken pituitary cells was investigated (King et al., 1989a) (Figure 1). E2, T, and progesterone inhibited LH release stimulated by GnRH, and the inhibitory effects required prolonged preincubation (24–48 h) with the steroids. E2 had no effect on binding of 125I-[His5,D-Tyr6]GnRH to a pituitary cell preparation. These findings suggest that the site of steroid modulation of GnRH action is distal to binding of GnRH to its receptor and that the inhibitory effects are exerted at intracellular sites (King et al., 1989a).

### By social interaction

Maney et al. (2007) played recordings of conspecific male song to laboratory-housed female white-throated sparrows and quantified the resulting rapid changes in LH as well as the induction of the immediate early gene Egr-1 in the GnRH neurons and MBH. Hearing song for 42 min induced LH release and Egr-1 expression in the MBH, but did not alter Egr-1 expression in GnRH neurons. The time course of LH release and the pattern of Egr-1 expression together suggest that song acts as a trigger to induce GnRH release. The Egr-1 response in the MBH was qualitatively distinguishable from the responses to either photostimulation or pharmacologically-induced LH release but seemed to involve overlapping neuronal populations (Maney et al., 2007).

## Role of GnIH and its receptor

GnIH was first isolated in the brain of the Japanese quail, *Coturnix japonica*, while searching for a novel RFamide peptide in birds. RFamide peptides, which have an Arg-Phe-NH_2_ motif at their C-terminus, were first discovered in invertebrate species in the late 1970s. The first RFamide peptide, Phe-Met-Arg-Phe-NH_2_ (FMRFamide), was a cardioexcitatory molecule isolated from the ganglia of the venus clam *Macrocallista nimbosa* (Price and Greenberg, 1977). Since then, numerous RFamide peptides that act as neurotransmitters, neuromodulators, and peripheral hormones have been identified in various invertebrates, including cnidarians, nematodes, annelids, molluscs, and arthropods. Subsequent immunohistochemical studies in vertebrates suggested the presence of RFamide peptides in the central nervous system. Because some FMRFamide-ir neurons projected close to the pituitary gland, it was suggested that the immunoreactive substance may regulate pituitary function in vertebrates.

In 2000, Tsutsui et al. isolated a novel RFamide peptide from 500 Japanese quail brains by HPLC and a competitive enzyme-linked immunosorbent assay using an antibody raised against the dipeptide Arg-Phe-NH_2_ (Tsutsui et al., 2000). The isolated peptide had a novel dodecapeptide structure, SIKPSAYLPLRFamide. Its C-terminus was identical to chicken LPLRFamide that was reported to be the first RFamide peptide isolated in vertebrates (Dockray et al., 1983), which may be a degraded fragment of the dodecapeptide. Because the isolated peptide was localized in the hypothalamo-hypophysial system, and shown to decrease gonadotropin release from cultured quail anterior pituitary glands, this RFamide peptide was named GnIH (Tsutsui et al., 2000) (Figure 1).

Following the isolation of GnIH from quail brain, a cDNA that encoded the GnIH precursor polypeptide was identified (Satake et al., 2001). The translated GnIH precursor consisted of 173 amino acid residues that encoded one GnIH and two GnIH-related peptides (GnIH-RP-1 and GnIH-RP-2), all possessing an LPXRFamide (X = L or Q) sequence at their C-termini. These peptide sequences were flanked by a glycine C-terminal amidation signal and a single basic amino acid on each end as an endoproteolytic site. GnIH-RP-2 was also identified as a mature peptide by mass spectrometry in quail (Satake et al., 2001). GnIH peptides were further isolated as mature peptides in European starlings (Ubuka et al., 2008a) and zebra finch (Tobari et al., 2010) within the class of birds (for reviews, see Tsutsui and Ukena, 2006; Tsutsui et al., 2006, 2010a,b, 2012; Ubuka et al., 2008b, 2012c; Tsutsui, 2009; Tsutsui and Ubuka, 2013).

To elucidate the mode of action of GnIH in birds, Yin et al. (2005) identified the GnIH receptor (GnIH-R) in quail diencephalon and characterized its expression and binding activity (Yin et al., 2005). First, a cDNA encoding a putative GnIH-R was cloned using PCR primers designed from the sequence of GPR147, a specific receptor for RFamide related peptide that is the orthologous peptide family of GnIH (Hinuma et al., 2000; Ubuka et al., 2009b,c, 2012a). The crude membrane fraction of COS-7 cells transfected with the putative GnIH-R cDNA specifically bound GnIH and GnIH-RPs in a concentration-dependent manner, indicating that GPR147 is a GnIH-R candidate (Yin et al., 2005). GnIH-R also bound with high affinity to GnIH, GnIH-RPs, and RFRPs, which have LPXRFamide (X = L or Q) motif at their C-termini. In contrast, non-amidated GnIH failed to bind the receptor. Accordingly, the C-terminal LPXRFamide (X = L or Q) motif seems to be critical for its binding to GnIH-R. Although GnIH-R bound GnIH and GnIH-RPs with similar affinities in this study, further studies are required to investigate if GnIH and GnIH-RPs work additively or synergistically to achieve their effects on the cells that express GnIH-R.

Shimizu and Bédécarrats (2010) showed in the chicken pituitary gland that GnIH-R mRNA levels fluctuate in an opposite manner to GnRH-R-III, a pituitary specific form of GnRH receptor (Shimizu and Bédécarrats, 2006; Joseph et al., 2009), with higher level of GnIH-R mRNA and lower level of GnRH-R-III observed during inactive and active reproductive stages, respectively (Shimizu and Bédécarrats, 2010). They also demonstrated that the chicken GnIH-R signals by inhibiting cAMP production, most likely by coupling to G_αi_. They also showed that this inhibition is sufficient to significantly reduce GnRH-induced cAMP responsive element (CRE) activation in a dose-dependent manner, and that the ratio of GnRH/GnIH receptors was a significant factor. From these results they proposed that in avian species, sexual maturation is characterized by a change in GnIH/GnRH receptor ratio, resulting in a switch in pituitary sensitivity from inhibitory, involving GnIH, to stimulatory, involving GnRH. In turn, decreasing GnIH-R signaling, combined with increasing GnRH-R-III signaling, results in significant increases in CRE activation, possibly initiating gonadotropin synthesis (Shimizu and Bédécarrats, 2010). Son et al. (2012) intensively studied the intracellular cell signaling mechanism of GnIH using immortalized mouse gonadotrope cell line (LβT2 cells). It was demonstrated that GnIH inhibits GnRH induced gonadotropin subunit gene transcriptions by inhibiting adenylate cyclase/cAMP/PKA-dependent ERK pathway in LβT2 cells (Son et al., 2012).

Although a dense population of GnIH neuronal cell bodies was only found in the paraventricular nucleus (PVN) of the hypothalamus, GnIH-ir neuronal fibers were widely distributed in the diencephalic and mesencephalic regions in birds (Ukena et al., 2003; Ubuka et al., 2008a, 2012b). Thus, it was hypothesized that GnIH may participate not only in the regulation of pituitary function, but also in behavioral and autonomic mechanisms in birds. Double-label immunocytochemistry showed GnIH axon terminals on GnRH1 and GnRH2 neurons in the starling brain, which is in agreement with an earlier study on house sparrows (Bentley et al., 2003; Ubuka et al., 2008a). *In situ* hybridization of starling GnIH-R mRNA combined with GnRH immunocytochemistry further showed the expression of GnIH-R mRNA in GnRH1 and GnRH2 neurons (Ubuka et al., 2008a) suggesting that GnIH modifies the activity of GnRH1 and GnRH2 neurons in the brain (Figure 1). GnRH2 enhances copulation solicitation in estrogen-primed female white-crowned sparrows exposed to the song of males (Maney et al., 1997). Because of the putative contact of GnIH neurons with GnRH2 neurons in white-crowned sparrows (Bentley et al., 2003), Bentley et al. (2006) investigated the effect of GnIH administration on copulation solicitation in females of this species. A centrally-administered physiological dose of GnIH inhibited copulation solicitation in estrogen-primed female white-crowned sparrows exposed to the song of males (Bentley et al., 2006) (Figure 1).

To identify the mechanism of GnIH neurons in the regulation of behavior, Ubuka et al. (2012b) investigated the effect of RNA interference (RNAi) of the GnIH gene on the behavior of white-crowned sparrows, a highly social songbird species. Administration of small interfering RNA against GnIH precursor mRNA into the third ventricle of male and female birds reduced resting time, spontaneous production of complex vocalizations, and stimulated brief agonistic vocalizations. GnIH RNAi further enhanced song production of short duration in male birds when they were challenged by playbacks of novel male songs. These behaviors resembled those of breeding birds during territorial defense. The overall results suggested that GnIH gene silencing induces arousal. In addition, the activities of male and female birds were negatively correlated with GnIH mRNA expression in the PVN. The density of GnIH neuronal fibers in the ventral tegmental area was decreased by GnIH RNAi treatment in female birds, and the number of GnRH neurons that received close appositions of GnIH neuronal fiber terminals was negatively correlated with the activity of male birds. In summary, GnIH may decrease arousal level resulting in the inhibition of specific motivated behavior, such as in reproductive contexts (Ubuka et al., 2012b, 2013) (Figure 1).

A dense population of GnIH-ir fibers at the median eminence in quail (Tsutsui et al., 2000; Ukena et al., 2003; Ubuka et al., 2003) as well as in other birds (Bentley et al., 2003; Osugi et al., 2004; Ubuka et al., 2008a) suggested a direct action of GnIH in the regulation of pituitary function in birds (Figure 1). The fact that GnIH inhibits synthesis and/or release of gonadotropins from cultured quail and chicken anterior pituitary gland provides strong support for this function (Tsutsui et al., 2000; Ciccone et al., 2004; Maddineni et al., 2008). Peripheral administration of GnIH also inhibits gonadotropin synthesis and/or release in birds (Osugi et al., 2004; Ubuka et al., 2006). On the other hand, it was suggested that GnIH may not act directly on the pituitary in some birds, because there are relatively few or no GnIH-ir fibers in the median eminence of Rufous-winged sparrows (Small et al., 2007)—although the anterior pituitary gland of this species expresses mRNA for GnIH-R (McGuire, Deviche and Bentley, unpublished data).

Maddineni et al. (2008) characterized the expression of GnIH-R mRNA and protein in the chicken pituitary gland. GnIH-R-ir cells were identified in the chicken pituitary gland cephalic and caudal lobes and they were co-localized with LHβ mRNA or FSHβ mRNA containing cells. GnIH treatment significantly decreased LH release from anterior pituitary gland slices collected from sexually immature, but not from sexually mature chickens. Taken together, GnIH-R protein expressed in FSHβ or LHβ mRNA containing cells is likely to mediate the inhibitory effect of GnIH on LH and FSH secretion (Maddineni et al., 2008) (Figure 1).

To clarify the functional significance of GnIH in the control of avian reproduction, Ubuka et al. (2006) investigated the action of GnIH on the HPG axis in male quail. It is generally accepted that in avian species LH stimulates the formation of T by Leydig cells. FSH and T stimulate growth, differentiation and spermatogenetic activity of the testis (Follett, 1984; Johnson, 1986). LH is a protein complex, which is made of gonadotropin common α and LHβ subunits, whereas FSH is a complex of gonadotropin common α and FSHβ subunits. Peripheral administration of GnIH to mature quail via osmotic pumps for 2 weeks decreased the expression of gonadotropin common α and LHβ subunit mRNAs in the pituitary. Concentrations of plasma LH and T were also decreased dose-dependently. Furthermore, administration of GnIH to mature birds induced testicular apoptosis and decreased spermatogenetic activity in the testis. In immature birds, daily administration of GnIH for 2 weeks suppressed testicular growth and the rise in the concentration of plasma T. An inhibition of molt by juveniles also occurred after GnIH administration. These results show that GnIH may inhibit gonadal development and maintenance and also sexual development of birds by decreasing the synthesis and release of gonadotropins (Ubuka et al., 2006) (Figure 1).

## Regulation of GnIH and GnIH-R

### By melatonin

Based on reports showing inhibitory effects of melatonin on the reproductive activities of quail (Ohta et al., 1989; Guyomarc'h et al., 2001), chicken (Rozenboim et al., 2002) and considering GnIH's inhibitory effects on the secretion of gonadotropins, Ubuka et al. (2005) hypothesized that melatonin may be involved in the induction of GnIH expression, thus influencing gonadotropin secretion. Px plus Ex decreased the expression of GnIH precursor mRNA and the content of mature GnIH peptide in the hypothalamus and melatonin administration to Px plus Ex birds caused a dose-dependent increase in the expression of GnIH precursor mRNA and the production of mature peptide. The mRNA of Mel_1c_, a melatonin receptor subtype, was expressed in GnIH-ir neurons in the PVN, and autoradiography of melatonin receptors revealed specific binding of melatonin in the PVN. Accordingly, melatonin appears to act directly on GnIH neurons through its receptor to induce expression of GnIH (Ubuka et al., 2005) (Figure 1).

Chowdhury et al. (2010) further investigated the role of melatonin in the regulation of GnIH release. Melatonin administration dose-dependently increased GnIH release from hypothalamic explants *in vitro*. GnIH release during the dark period was greater than that during the light period in explants from quail exposed to long day photoperiods. Conversely, plasma LH concentration decreased during the dark period. GnIH release increased under short day photoperiods, when the duration of nocturnal secretion of melatonin increases. These results indicate that melatonin plays a role in stimulating not only GnIH expression but also GnIH release, thus reducing plasma LH concentration in scotophase (Chowdhury et al., 2010) (Figure 1).

### By stress

Stress leads to reproductive dysfunction in many species. Calisi et al. (2008) hypothesized that stress effects upon reproduction are mediated via the hypothalamic GnIH system in birds. They examined the effects of capture-handling stress in the hypothalamus of male and female adult house sparrows. There were more GnIH-ir neurons in fall birds versus those sampled in the spring, and a significant increase in GnIH-ir neurons was seen in stressed birds in spring. These data imply an influence of stress upon the GnIH system that changes over the annual cycle of reproduction (Calisi et al., 2008) (Figure 1).

### By sex steroids

Maddineni et al. (2008) characterized the expression of GnIH-R mRNA and protein in the chicken pituitary gland, and determined whether sexual maturation and gonadal steroids influence pituitary GnIH-R mRNA abundance. GnIH-R mRNA quantity was significantly higher in the pituitaries of sexually immature chickens relative to sexually mature chickens. Estradiol or a combination of estradiol and progesterone treatment caused a significant decrease in pituitary GnIH-R mRNA quantity relative to vehicle controls. These results suggest that GnIH-R gene expression is down-regulated in response to a surge in circulating estradiol and progesterone levels as the chicken undergoes sexual maturation to allow gonadotropin secretion (Maddineni et al., 2008) (Figure 1).

### By social interaction

Calisi et al. (2011) experimentally manipulated nesting opportunities for pairs of European starlings and examined brain GnIH mRNA and GnIH content, as well as GnRH content and plasma T and corticosterone. By limiting the number of nest boxes per enclosure and thus the number of social pairing and nesting opportunities, they observed that birds with nest boxes had significantly fewer numbers of GnIH peptide-producing cells than those without nest boxes and this relationship changed with breeding stage. GnRH content, T and corticosterone did not vary with nest box ownership. These data suggest that GnIH may serve as a modulator of reproductive behaviors without dramatically changing the HPG axis in response to social environment (Calisi et al., 2011).

## Conclusion

As we reviewed here, the reproductive physiology and behavior of birds are exquisitely regulated by various environmental signals to maximize fitness. Social interactions within the species also have dramatic effects on reproductive physiology and behavior of birds. Indispensable factors for reproduction, such as the establishment of territories, interactions with the opposite sex, and food availability seem to be very important to the timing of breeding. We attempted to understand how environmental information and social interactions are integrated to regulate the HPG axis in birds. We concentrated on the two hypothalamic neuropeptide system (GnRH and GnIH neuronal systems) in this review to understand a central mechanism regulating seasonal reproduction (Figure 1). There are still many missing links in understanding how GnRH and GnIH neuronal systems integrate various external and internal environmental information and control gonadotropin secretion to time seasonal reproduction in birds. Environmental and social milieus fluctuate seasonally in the wild. Accordingly, coordination of various known and unknown neural and hormonal mechanisms may be needed to translate various environmental information and social interactions to produce appropriately-timed changes in the reproductive physiology and behavior of birds.

### Conflict of interest statement

The authors declare that the research was conducted in the absence of any commercial or financial relationships that could be construed as a potential conflict of interest.
